# From Grapheme to Phonological Output: Performance of Adults Who Stutter on a Word Jumble Task

**DOI:** 10.1371/journal.pone.0151107

**Published:** 2016-03-10

**Authors:** Megann McGill, Harvey Sussman, Courtney T. Byrd

**Affiliations:** Department of Communication Sciences and Disorders, The University of Texas at Austin, Austin, Texas, United States of America; University College London, UNITED KINGDOM

## Abstract

**Purpose:**

The purpose of the present study was to extend previous research by analyzing the ability of adults who stutter to use phonological working memory in conjunction with lexical access to perform a word jumble task.

**Method:**

Forty English words consisting of 3-, 4-, 5-, and 6-letters (*n* = 10 per letter length category) were randomly jumbled using a web-based application. During the experimental task, 26 participants were asked to silently manipulate the scrambled letters to form a real word. Each vocal response was coded for accuracy and speech reaction time (SRT).

**Results:**

Adults who stutter attempted to solve fewer word jumble stimuli than adults who do not stutter at the 4-letter, 5-letter, and 6-letter lengths. Additionally, adults who stutter were significantly less accurate solving word jumble tasks at the 4-letter, 5-letter, and 6-letter lengths compared to adults who do not stutter. At the longest word length (6-letter), SRT was significantly slower for the adults who stutter than the fluent controls.

**Conclusion:**

Results of the current study lend further support to the notion that differences in various aspects of phonological processing, including vision-to-sound conversions, sub-vocal stimulus manipulation, and/or lexical access are compromised in adults who stutter.

## Introduction

Stuttering is a multifactorial communication disorder that interrupts the forward flow of speech production (e.g., [1], [2], [3], [4]). There are significant data to suggest deficits in phonological working memory may be one of the factors that contribute to the difficulties persons who stutter have establishing and/or maintaining fluent speech, particularly when presented with cognitively demanding tasks (for review, see [5], [6]; cf., [7]). Reduced speed and accuracy in aspects of phonological working memory, including phonological encoding and sub-vocal rehearsal, have been observed in children and adults who stutter in vocal (e.g., [8], [9], [10], [11], [12]) and nonvocal tasks (e.g., [13, [14], [15], [16]). Additionally, neuroimaging results using diffusion tensor tractography and magnetoencephalography lend further support to the assumption that the phonological processing of persons who stutter differs from fluent controls (e.g., [17], [18], [19], [20], [21], [22], [23]).

### Phonological working memory

**Fig 1:**
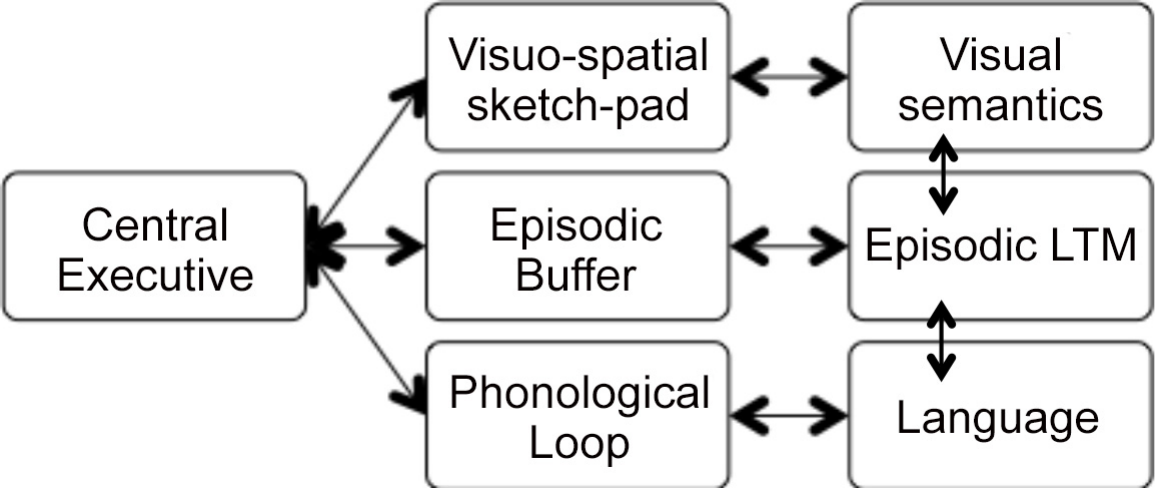
Adapted version of Baddeley’s theoretical model of working memory [24].

Theoretically, Baddeley’s [24] model creates a framework for understanding the contributions of phonological working memory to fluent speech production. Baddeley’s [24] model of working memory is comprised of the central executive and three supporting systems: (1) phonological loop, (2) visuospatial sketchpad, and (3) episodic buffer (Fig 1). The central executive is thought to support the retrieval and transfer of information from long-term memory to short-term memory and vice versa. The phonological loop is comprised of the following two critical components: a phonological store and a sub-vocal rehearsal system. The phonological store facilitates the ability to hold material to be remembered in a phonological code. This phonological code is vulnerable to decay over time (i.e., trace will last approximately 2 seconds), hence the need for the sub-vocal rehearsal system. The sub-vocal rehearsal system is a silent verbal repetition process that refreshes the phonologically encoded material, allowing it to be preserved in memory for a longer period of time (>2 seconds). The second system, the visuospatial sketchpad, stores and manipulates visual and spatial information for retention. The third and final component of Baddeley’s model, the episodic buffer, passively binds information from various distinct sources (i.e., phonological, visual, spatial) into chunks or episodes for transference to the central executive [24].

### Nonword repetition, phoneme elision, and nonword reading tasks

A variety of experimental paradigms have been completed with persons who stutter to enhance our understanding of the contribution of various subsystems of phonological working memory to stuttered speech. Nonword repetition tasks have been used to explore phonological working memory in adults who stutter (e.g., [8], [11], [12], [13], [25]). Nonword repetition tasks tap into phonological working memory by requiring phonological encoding and, potentially, sub-vocal rehearsal of a novel phonological string prior to repetition without the influence of semantic information. Results from several nonword repetition studies have demonstrated that adults who stutter are less accurate repeating nonwords than their fluent peers (e.g., [8], [11], [12], [13], [25]). Phoneme elision tasks using nonword stimuli have also recently been implemented to investigate the abilities of persons who stutter to phonologically encode, sub-vocally rehearse, and manipulate novel phonological strings prior to producing a novel word.

Ludlow et al. [25] completed one of the first investigations of nonword repetition with adults who stutter. Ten adults who do and do not stutter repeated two, 4-syllable nonwords multiple times. Adults who stutter demonstrated significantly decreased accuracy compared to fluent controls, even when provided with the opportunity to repeat the nonwords. The authors interpreted these findings to indicate that adults who stutter present with phonological encoding deficits as compared to adults who do not stutter. Additional research has also supported the finding of differences in phonological processing specific to adults who stutter.

Byrd et al. [8] employed a nonword repetition task as well as a phoneme elision task. Fourteen adults who stutter and 14 adults who do not stutter listened to 48 nonwords, were provided multiple attempts at production, and then were required to repeat the target nonword with a sound missing. There was no difference in performance accuracy on the phoneme elision task between adults who stutter and fluent controls; this task was equally challenging for both talker groups. However, for the nonword repetition task, results showed repetition accuracy was comparable for adults who do and do not stutter for their repetitions of 2–4 syllable words, but the adults who stutter required a greater mean number of attempts before accurate repetition of 7-syllable words. The authors attributed the significant findings for the nonword repetition task to suggest that there is a deficit in the sub-vocal rehearsal system of adults who stutter, which is highlighted when the required productions are at lengths that are more challenging to recall.

Additionally, Sasisekaran [11] tested behavioral and kinematic responses of 18 adults who do and do not stutter during nonword repetition and nonword reading tasks. Talker groups did not differ in their nonword repetition accuracy, but adults who stutter demonstrated a higher percentage of errors in nonword reading than fluent controls. However, the stimuli used in their nonword repetition task were one to four syllables in length while the stimuli for their nonword reading task were either six or 11-syllables long. Thus, it is possible that the nonwords used for the repetition task were not sufficiently complex to tax the phonological working memory of the adults who stutter in a manner that would yield the talker group differences previously observed by Byrd et al. [8].

More recently, Sasisekaran and Weisberg [12] explored the nonword repetition accuracy of 10 adults who stutter when auditorily presented with nonword stimuli that varied by complexity and phonotactic constraint. Adults who stutter demonstrated decreased accuracy repeating complex nonwords compared to their fluent peers. Additionally, adults who stutter exhibited significant practice effects as measured by reduced movement variability for 3-syllable stimuli, but motoric variability persisted for the longer 4-syllable words. The authors attributed talker group differences in nonword repetition accuracy to deficits in phonemic encoding and/or speech motor processes for adults who stutter.

### Vocal versus nonvocal tasks

Sasisekaran, DeNil, Smyth, and Johnson [26] explored nonvocal phonological encoding of adults who stutter using a phoneme monitoring task. Ten adults who stutter and 12 adults who do not stutter completed phoneme monitoring tasks during a silent naming condition and a perception condition. During the silent naming condition, participants pressed a button to indicate whether or not a target sound was present when they silently named a picture. In the perception condition, participants silently indicated whether or not a target sound was heard during auditorily presented items. Results revealed adults who stutter were significantly slower in phoneme monitoring during the silent naming task. The authors suggested that these results support the notion that adults who stutter exhibit phonological encoding deficits during silent encoding and segmenting of phonological units as compared to fluent controls.

In an attempt to extend past findings beyond the restriction to vocal performance, Byrd, McGill, and Usler [13] explored the vocal and nonvocal nonword repetition and phoneme elision abilities of adults who stutter compared to adults who do not stutter. Nonvocal conditions were added in this study in order to avoid possible confounds related to errors of motor execution. In the vocal nonword repetition task, participants listened to and repeated two sets of 4- and 7-syllable nonwords (*n* = 12 per set; 24 total). For the nonvocal nonword repetition task, participants listened to the same set of 24 nonwords and silently identified each target nonword from a subsequent set of three nonwords. In the vocal phoneme elision task, participants repeated the 24 nonwords with a designated phoneme eliminated. Similar to the nonvocal nonword repetition task, in the nonvocal phoneme elision task, participants listened to phoneme elision instructions and silently identified which subsequently presented nonword accurately fulfilled the condition. Adults who stutter produced significantly fewer accurate initial vocal productions of 7-syllable nonwords compared to adults who do not stutter. There were no participant group differences for the silent identification of nonwords, but both groups required significantly more attempts to accurately silently identify 7-syllable nonwords as compared to 4-syllable nonwords. For the vocal phoneme elision condition, adults who stutter were significantly less accurate than adults who do not stutter in their initial production and required significantly more attempts to accurately produce 7-syllable nonwords with a designated phoneme eliminated. This group difference was also significant for the nonvocal phoneme elision condition for both 4- and 7-syllable nonwords. The authors suggested performance differences on these vocal and nonvocal tasks further support the notion that phonological working memory may contribute to the difficulty adults who stutter have establishing and/or maintaining fluent speech.

### Paradigm Selection

Taken together, results from multiple experimental investigations support the idea that phonological working memory, particularly the subsystems that Baddeley [24] describes as phonological encoding and/or sub-vocal rehearsal, may be uniquely compromised in persons who stutter. To further extend previous investigations of phonological working memory in adults who stutter that have been restricted to auditory input, the current investigation utilizes visual stimuli in a word jumble paradigm. This methodological distinction includes the additional task demands of (1) visual decoding of letter strings, (2) grapheme-to-sound conversions, and (3) lexical search/retrieval operations.

**Fig 2:**
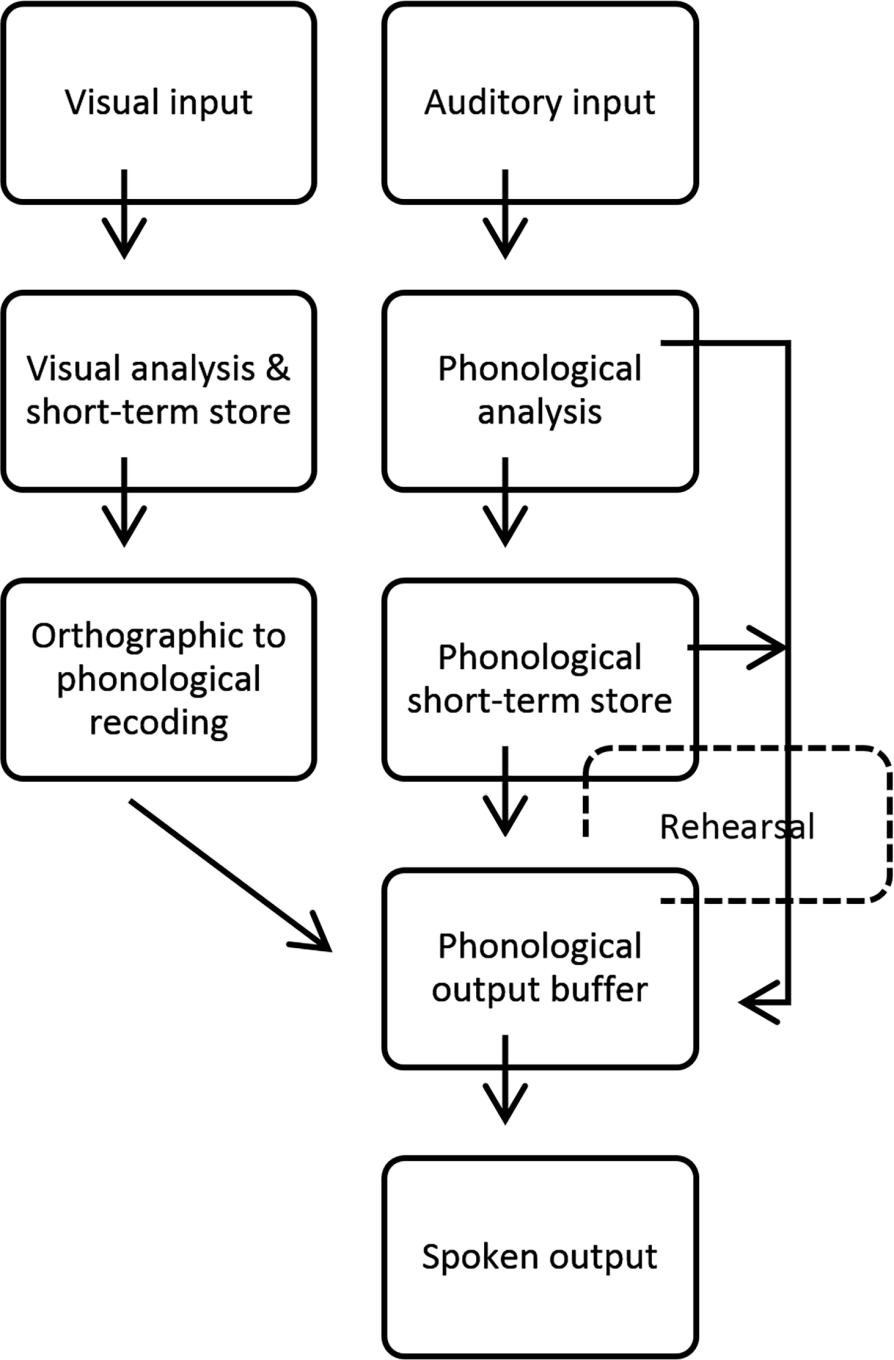
Adapted version of Baddeley’s [24] proposed structure of the phonological loop with visual and/or auditory input.

Baddeley [24] describes a model of the phonological loop that accounts for both visual and auditory input eventually arriving at the phonological output buffer, which may either lead to sub-vocal rehearsal or spoken output. Baddeley [24] posits that visually presented material is decoded at the level of visual analysis then transferred from an orthographic to a phonological code to be temporarily stored within the phonological output buffer (See Fig 2). While the phonological code is held within the phonological output buffer, the central executive facilitates a rapid retrieval process from the lexicon. In the current experimental paradigm, participants were asked to reorder the orthographic-phonological code through a nonvocal manipulation process. This silent manipulation assists in the trial and error reordering of the visual and phonological information multiple times prior to finally supplying a complete, lexically appropriate solution to the phonological output buffer which will then be executed through spoken word production. Thus, successful performance on this meta-linguistic task requires neural facility with several phonologically based lexical skill sets, including phonotactic sensitivities, visualization of re-ordered lexical strings, and matching those manipulations with lexical reality.

### Purpose and Related Predictions

The purpose of the present study is to analyze the ability of adults who stutter to use phonological working memory in conjunction with lexical access as measured by performance on a word jumble task. In contrast to previous investigations of phonological working memory that have utilized auditory input only (e.g., nonword repetition, phoneme elision), the current paradigm presents visual stimuli that must then be translated into a phonological code to be reordered. Specifically, the word jumble paradigm requires participants to (1) initially encode a visually presented, randomly ordered orthographic string, and subsequently, (2) mentally manipulate, in trial and error fashion, various phonologically possible arrangements, that (3) trigger the emergence and recognition of a real word. We predict that adults who stutter will demonstrate increased response times and decreased accuracy in rearranging jumbled stimuli to arrive at real words compared to adults who do not stutter.

## Method

### Participants

The current study and consent form documentation was approved by the University of Texas at Austin’s Institutional Review Board (IRB approval number 2014-03-0068) and written, informed consent was obtained for each participant. Participants met the following inclusionary criteria: (a) self-reported native English speaker or English speaker with native competency; (b) 18 years of age or older; (c) no present or prior history of speech, reading, and/or language disorders (with the exception of stuttering for the adults who stutter); and (d) no neurological, social, emotional, or psychiatric disturbances. Participants were 26 adults who do (*n* = 13; *M* = 28.15 years; range = 19–42; *n* = 5 female; *n* = 8 males) and do not stutter (*n* = 13; *M* = 28.5 years; range = 19–42; *n* = 5 female; *n* = 8 males) matched for age (±3 years), gender, handedness, and education-level. All 13 of the adults who stutter had reportedly received prior speech therapy for stuttering. See Table 1 for participant descriptions.

**Table 1:** Participant characteristics for adults who do not stutter (AWNS) and adults who stutter (AWS).

Participant	Age	Handedness	Gender	Education Level	Severity	PPVT-4	EVT-2	CTOPP-PE	CTOPP-BW	CTOPP-RDN	CTOPP-NWR
1	22	Right	Male	College	ML	123	114	9	13	10	13
2	42	Right	Female	Graduate School	SV	105	105	4	10	9	8
3	19	Right	Male	College	ML	117	118	11	12	11	13
4	28	Left	Female	College	SV	106	113	11	6	10	9
5	42	Right	Male	Masters	MOD	107	106	10	9	9	11
6	27	Right	Male	College	VS	86	90	9	4	6	4
7	35	Right	Female	College	ML	107	110	7	11	2	10
8	31	Left	Female	College	ML	114	104	11	10	11	9
9	27	Right	Male	College	ML	96	102	11	10	20	7
10	20	Right	Male	College	VML	113	121	9	10	9	8
11	21	Right	Male	College	VML	127	115	8	10	7	11
12	20	Right	Female	College	ML	91	97	10	13	7	13
13	32	Right	Male	College	VS	109	118	8	10	9	10
14	21	Right	Male	College	N/A	108	102	10	11	12	13
15	25	Left	Female	College	N/A	117	120	12	14	8	12
16	23	Right	Male	College	N/A	128	116	4	11	12	12
17	32	Right	Female	College	N/A	136	120	12	14	9	14
18	27	Right	Male	College	N/A	109	112	11	12	12	14
19	19	Right	Female	College	N/A	98	104	12	12	12	14
20	35	Right	Male	College	N/A	107	104	11	14	9	10
21	27	Right	Male	College	N/A	113	116	11	13	12	13
22	19	Right	Male	College	N/A	126	116	11	10	9	12
23	34	Left	Female	College	N/A	113	104	11	13	16	13
24	39	Right	Male	Masters	N/A	110	115	11	10	12	11
25	25	Right	Male	College	N/A	129	136	12	13	12	11
26	42	Right	Female	Graduate School	N/A	103	113	9	13	10	12

Peabody Picture Vocabulary Test-Fourth Edition (PPVT-4): Standard score (M = 100, SD = 15). Expressive Vocabulary Test-Second Edition (EVT-2): Standard score: (M = 100, SD = 15). Comprehensive Test of Phonological Processing (CTOPP) subtests: Standard score: (M = 10, SD = 2). Severity: VM = very mild, ML = mild, MOD = moderate, SV = severe, VS = very severe.

### Classification and Inclusion Criteria

#### Speech, language, hearing, and vision measures

All participants passed a bilateral pure tone hearing screening at 20dB for 1000, 2000, and 4000Hz [27]. All participants met the 20/20 criterion on a near visual acuity test [28]. Additionally, all participants were administered the following pre-experimental standardized language and speech measures: *Peabody Picture Vocabulary Test–Fourth Edition* (PPVT-4; [29]), *Expressive Vocabulary Test–Second Edition* (EVT-2; [30]), *Comprehensive Test of Phonological Processing* subtests Phoneme Elision, Blending Words, Rapid Digit Naming, and Nonword Repetition (CTOPP; [31]). All participants reported no past or present diagnosis of a speech, language, and/or hearing impairment with exception of stuttering for the participants who stutter. All participants reported no present or prior diagnosis of dyslexia and no present or prior general or specific difficulties with reading. All participants also reported no significant neurological or cognitive deficits as part of their medical history.

#### Talker group classification

A participant was considered an adult who stutters if he or she self-reported as a person who stutters, if the participant had received a formal diagnosis of stuttering from a certified speech-language pathologist, if the participant exhibited three or more stuttering-like disfluencies (e.g., sound/syllable repetitions, sound prolongations, whole-word repetitions) per 300-word speech sample [32], and if the participant presented with an overall score of 10 or higher on the *Stuttering Severity Instrument for Children and Adults- Third Edition* (SSI-3; [33]). A participant was classified as an adult who does not stutter if he or she exhibited fewer than two stuttering-like disfluencies [32] per 300-word sample and a score of less than 10 on the SSI-3 [33], and if the person reported they had no past or present diagnosis of stuttering.

#### Stuttering severity

Stuttering severity was determined from video recorded conversational and reading samples with consideration of duration of disfluent moments and physical concomitants. Each participant’s samples were analyzed by the first author using the SSI-3 [33]. Of the 13 participants who were classified as adults who stutter, two received a rating of very mild stuttering, six received ratings of mild stuttering, one received a rating of moderate stuttering, two received a rating of severe stuttering, and two received a rating of very severe stuttering.

Inter-rater reliability of stuttering severity for speech samples was determined by the first author and an undergraduate research assistant trained in disfluency count analysis. Eight of the 26 participants (30%; 4 adults who stutter, 4 adults who do not stutter) were selected at random to determine inter-rater and intra-rater reliability. For adults who stutter, inter-rater reliability was within two points on the SSI-3 for the four participants. Thus, the reliability was found to be Kappa = 0.94. There was 100% agreement for the severity ratings for all four participants who do not stutter, with no stuttering-like disfluencies noted during the conversational samples.

#### Word Jumble familiarity

Participants self-reported experience with word jumble games in books, online, or in the newspaper prior to beginning the experimental task. Participants reported their frequency of completion of word jumble tasks within the last year on a 5-point Likert scale (i.e., 1 never, 2 = rarely, 3 = occasionally, 4 = a moderate amount, 5 = a great deal). All 26 participants reported scores of 1 or 2, indicating infrequent exposure to word scramble activities.

### Stimuli Development

Forty English words consisting of 3-, 4-, 5-, and 6-letters (*n* = 10 per letter length category) were selected from word lists developed by Snodgrass and Vanderwart [34] and Juhasz, Lai, and Woodcock [35]. Stimuli were controlled for age of acquisition and visual familiarity as described by Snodgrass and Vanderwart [34] and Juhasz et al. [35]. The mean age of acquisition for 3-letter words was 3.02 years. The mean age of acquisition for 4-letter words was 3.13 years. Mean age of acquisition for 5- letter words was 3.25 years and was calculated at 3.82 years for 6-letter words.

Each of the 40 words selected for use in the study was then randomly jumbled using a web-based application [36]. To generate the randomly jumbled word, the English spelling for each stimulus item was typed into the Word Scramble Maker. The application randomly transposed letters within each word to produce a scrambled, or jumbled, stimulus. The same scrambled stimulus of a word was used across all participants. See Table 2 for the word jumble stimuli used in the present study.

**Table 2:** Word jumble stimuli.

3-Letter Words	Jumbled Examples	5-Letter Words	Jumbled Examples
1. bed	edb	1. clock	lccko
2. fox	ofx	2. glove	eogvl
3. sun	nus	3. arrow	roarw
4. hat	ath	4. house	hseuo
5. key	eyk	5. camel	malec
6. fly	lfy	6. peach	epcha
7. and	dan	7. eagle	eaelg
8. car	rca	8. flute	ftule
9. gun	gnu	9. horse	erohs
10. nut	tun	10. ruler	rurle
4-Letter Words	Jumbled Examples	6-Letter Words	Jumbled Examples
1. drum	urdm	1. tomato	attoom
2. moon	oonm	2. anyone	yennoa
3. sock	ksco	3. jacket	cjteak
4. bear	arbe	4. hotdog	gdooht
5. vest	etsv	5. turtle	eturtl
6. iron	roni	6. subway	wbsyua
7. nail	inal	7. monkey	nkmeyo
8. lion	oiln	8. batboy	baytob
9. kite	eikt	9. finger	ngeifr
10. fish	fihs	10. uplift	lufpit

### Procedures

Pre-testing, including standardized measures of expressive language, receptive language, phonological processing, and stuttering severity were complete immediately preceding the administration of experimental tasks. Experimental tasks were completed in a quiet room over one session lasting approximately 30 minutes. Participants viewed stimuli on a 35” Dell computer monitor. The distance between the participant, seated on a chair, and the computer screen was approximately 18 inches. Stimuli were presented using 40 point, Arial, black font on a white background using Microsoft PowerPoint. Scrambled words were presented one at a time on the computer display. Participants were asked to silently manipulate the scrambled letters to form an English word. Participants were given one opportunity to verbally produce each unscrambled stimulus real word. After the participant successfully unscrambled the letter string to arrive at a word, or failed to produce a response, he or she manually advanced to the next scrambled stimulus by left-clicking the mouse. There was no time limit, but participants were encouraged to respond as quickly as possible to each stimulus.

Participants were read the following directions prior to beginning the task: “You will see a string of jumbled letters appear on the screen. Mentally manipulate or switch around the letters so that they form a real word. Verbally state your answer as soon as you have unscrambled the word. Each word jumble only has one correct answer. Please do not mouth, whisper or “think out loud.” You only have one opportunity to state your answer. After you have said the word, advance to the next slide by clicking the mouse. Do you have any questions?” One practice item preceded the experimental tasks. Participants were given the opportunity to ask questions after the practice item. No participants were observed moving their mouths, whispering, or thinking aloud during the experimental tasks.

For all participants, the stimuli were presented according to increasing complexity. Thus, participants were first presented with the 3-letter words, then the 4-letter words, followed by the 5-letter words, and finally ending with the most challenging, 6-letter words. The order of words within the letter-length classes was counterbalanced across participants in both groups.

### Coding

Participant responses were recorded by hand using a paper score sheet and digitally using a Shure SM58 microphone placed approximately six inches from the speaker’s mouth. Responses were also documented using a Sanyo VPC-HD1010 digital video recorder, an Olympus WS-321M digital voice recorder, and the record presentation function on Microsoft PowerPoint. Multiple recording methods were used to ensure that participants’ responses were recorded in the case of technical difficulty. Each response was coded for accuracy and speech reaction time (SRT). Participants’ attempts to unscramble the word were scored as either being 1) correct, 2) an error (e.g., stating “atom” given the jumbled stimulus “attoom”), or 3) no response (e.g., “I can’t figure it out.” or advancing to the next stimulus without attempting to answer). Stuttered productions and responses beginning with non-specific vocalizations (e.g., “uhm”, “er”) were excluded from further analysis. These exclusions occurred three times for adults who do not stutter and four times for adults who stutter. All responses for each participant were reviewed offline by the initial scorer and also by a trained research assistant to ensure accuracy in transcription and no discrepancies were found.

Participants’ SRTs, in milliseconds, were calculated by analyzing participants’ verbal responses using Praat software. The record function on Microsoft PowerPoint simultaneously created a new audio recording when each word jumble stimulus appeared on the screen. Audio files were exported from Microsoft PowerPoint and analyzed using Praat software. Thus, SRT was calculated from the beginning of the recording, which coincided exactly with the appearance of the visual stimulus, to the onset of the participant’s vocal response for each word jumble stimulus. The first author and a trained research assistant independently, visually inspected each of the waveforms and spectrograms of the participants’ responses to determine SRT for each word jumble stimulus. Inter-rater reliability was 90% and intra-rater reliability was 100%.

## Results

Statistical analyses were conducted using IBM SPSS Statistics Version 23. Alpha level was set at 0.05. Levene’s Test for Equality of Variances was completed for each analysis as well as Mauchly’s Test of Sphericity for each repeated measure analysis. One adult who stutters presented with SRT that was calculated at greater than two standard deviations above the mean for adults who stutter and was, thus, excluded from statistical analyses related to SRT in order meet the ANCOVA assumption of no outliers.

### Speech, language, and phonological testing

Independent *t*-tests conducted on the mean scores demonstrated that the performances of adults who stutter (*M* = 107.77; SD = 11.76) and adults who do not stutter (*M* = 115.15; SD = 11.35) did not significantly differ for receptive vocabulary; *t*(24) = 1.63, *p* = 0.116. The expressive vocabulary of the adults who stutter (*M* = 108.69; SD = 9.07) did not significantly differ from the adults who do not stutter (*M* = 113.69; SD = 9.21); *t*(24) = 1.395, *p* = 0.176. Likewise, no significant differences were found between the talker groups for CTOPP subtests Phoneme Elision (PE) or Rapid Digit Naming (RDN) (CTOPP-PE: adults who stutter *M* = 9.08, SD = 2.02; adults who do not stutter *M* = 10.54, SD = 2.15; *t*(24) = 1.79, *p* = 0.086; CTOPP-RDN: adults who stutter *M* = 9.23, SD = 4.04; adults who do not stutter *M* = 11.15, SD = 2.11; *t*(24) = 1.52, *p* = 0.142). Independent *t*-tests conducted on the mean scores demonstrated that the performances of adults who stutter and adults who do not stutter significantly differed on CTOPP Blending Words (BW: adults who stutter *M* = 9.85, SD = 2.51; adults who do not stutter *M* = 12.31, SD = 1.44; *t*(24) = 3.067, *p* = 0.005). Additionally, a *t*-test on the mean scores of CTOPP Nonword Repetition (NWR) revealed significant group differences. Levene’s test for equality of variances was found to be violated for the CTOPP NWR analysis *F*(1, 24) = 4.426, *p* = 0.046. Thus, a *t-*statistic not assuming homogeneity of variance was computed for CTOPP NWR scores for adults who stutter (*M* = 9.69, SD = 2.63) and adults who do not stutter (*M* = 12.38, SD = 1.26); *t*(17.25) = 3.33, *p* = 0.004. To confirm the assumptions of ANCOVA, no significant interactions between the covariates (i.e., CTOPP BW, CTOPP NWR) and Word Length or Talker Group variables were found; furthermore, variance inflation factor (VIF) for all variables was below 2.5, indicating no multicollinearity. Thus, performances on CTOPP NWR and CTOPP BW subtests were included in statistical models analyzing word jumble accuracy and SRT as covariates.

### Word jumble accuracy

**Fig 3:**
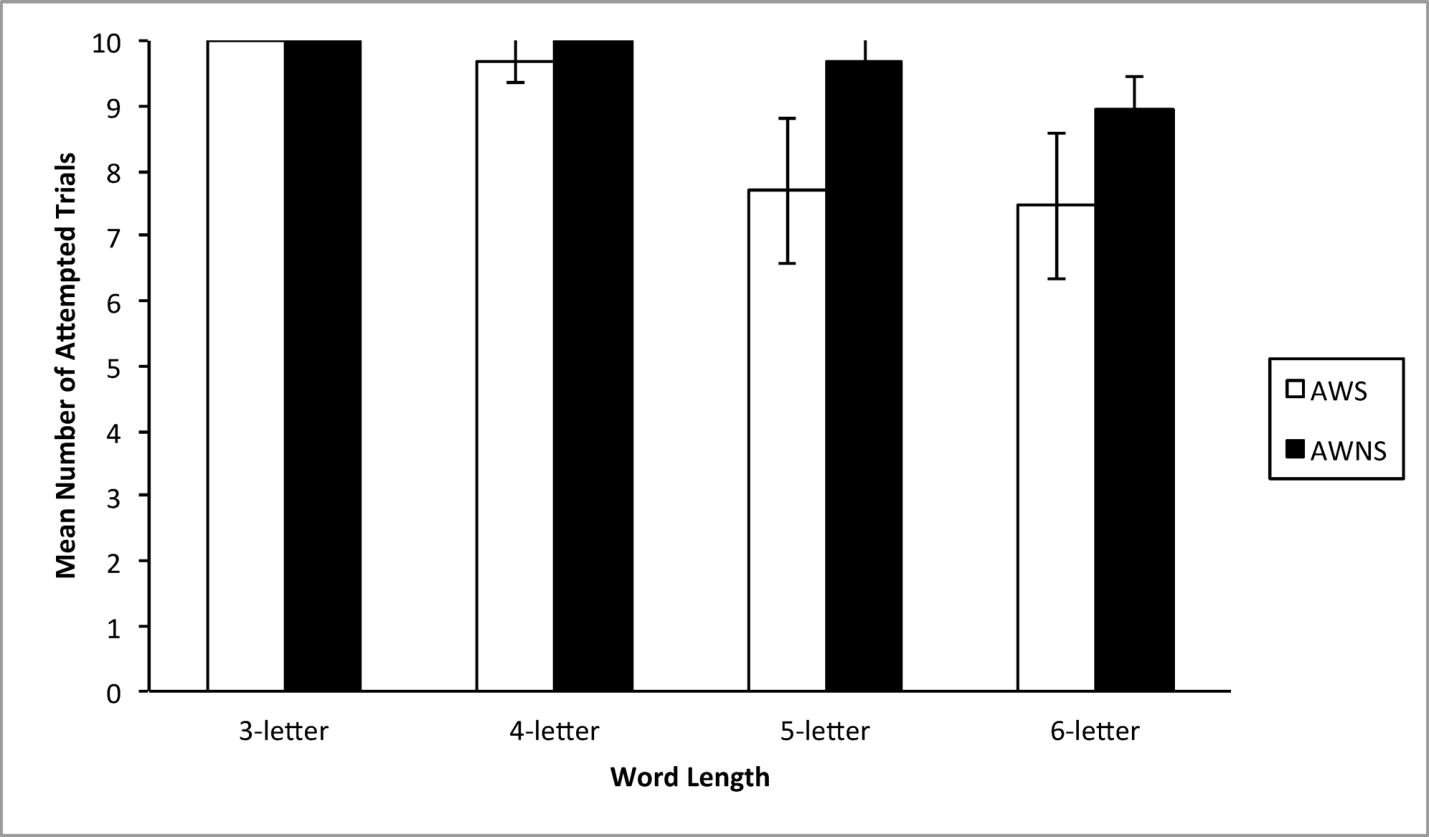
The mean number (+/- two standard errors) of attempted trials for each word jumble task across word-length classes for adults who stutter (AWS) and adults who do not stutter (AWNS).

As shown in Fig 3, all participants produced attempts to unscramble the 10 stimuli presented at the 3-letter length. At the 4-letter length, adults who stutter demonstrated fewer attempted trials to solve the word jumble stimuli compared to adults who do not stutter; however, this difference was not significant (t(12) = 1.760, p = 0.104), while controlling for CTOPP BW and CTOPP NWR. Adults who stutter presented with significantly fewer attempted trials compared to adults who do not stutter, controlling for CTOPP BW and CTOPP NWR, at the 5-letter (t(15.268) = 3.352, p = 0.04), and 6-letter lengths (t(17.075) = 2.354, p = 0.031). That is, adults who stutter attempted to solve significantly fewer word jumbles than adults who do not stutter at the 5- and 6-letter lengths.

**Fig 4:**
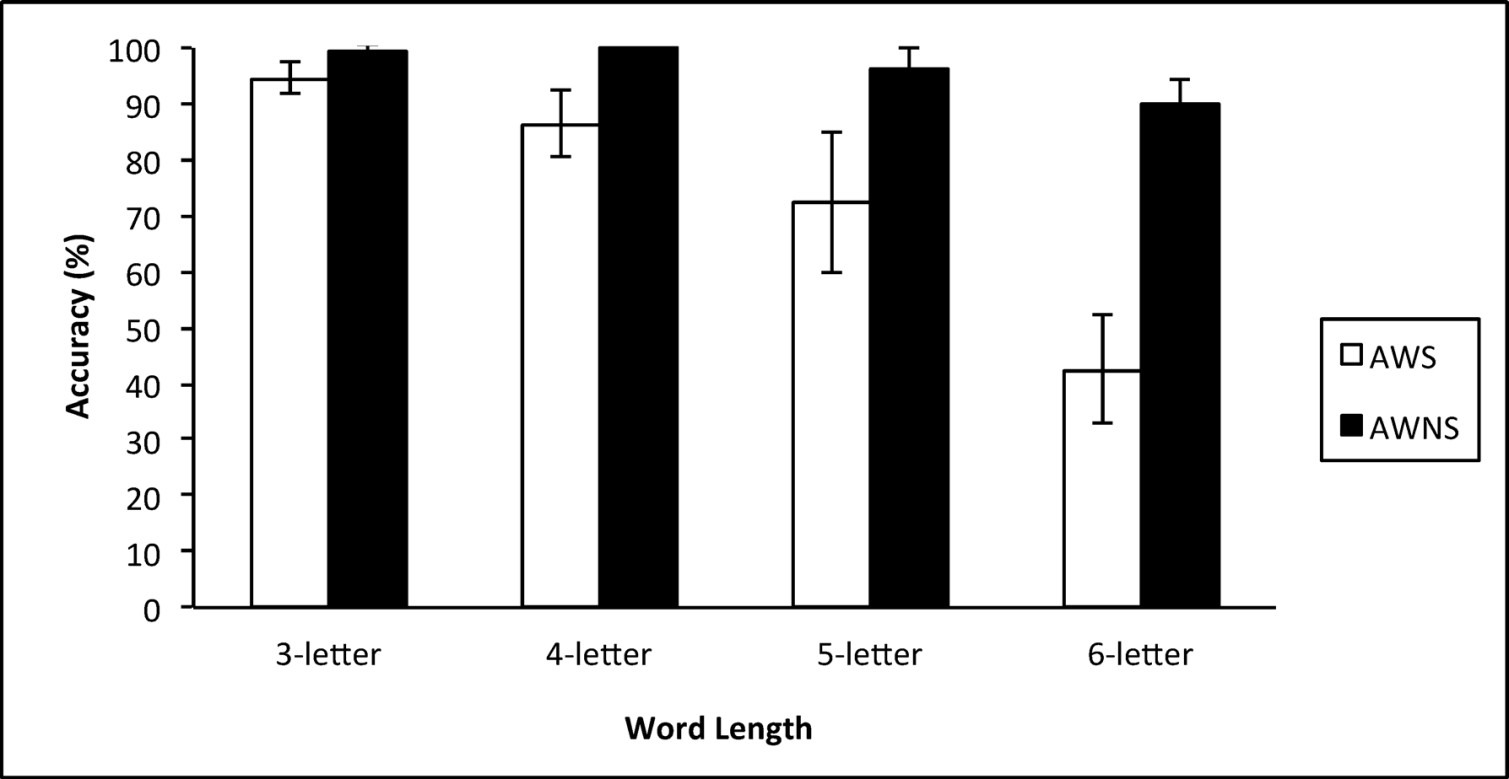
The mean percentage (+/- two standard errors) of accurate responses for each word jumble task across word-length classes for adults who stutter (AWS) and adults who do not stutter (AWNS).

A mixed-model repeated measures ANCOVA was conducted with the between-subjects factor of Talker Group (adults who stutter, adults who do not stutter), a within-subjects factor of Word Length (3-, 4-, 5-, 6-letter length), and CTOPP NWR and CTOPP BW performances as covariates. The dependent variable was the percentage of accurately unscrambled real word responses. Results revealed a significant between-subjects effect for Talker Group *F*(1,22) = 42.175, *p* ≤ 0.000, partial *η*^2^ = 0.657 and a significant interaction between Talker Group and Word Length *F*(3,22) = 12.762, *p* ≤ 0.000, partial *η*^2^ = 0.367. No significant main effect was noted for Word Length *F*(3,22) = 1.003, *p* = 0.397, partial *η*^2^ = 0.044. Decomposition of the interaction between Talker Group and Word Length, while controlling for CTOPP BW and CTOPP NWR, revealed a significant difference between adults who stutter and adults who do not stutter at the 4-letter (*t*(22) = 3.828, *p* ≤ 0.001, partial *η*^2^ = 0.400), 5-letter (*t*(22) = 2.70, *p* = 0.013, partial *η*^2^ = 0.249), and 6-letter lengths (*t*(22) = 7.113, *p* ≤ 0.0001, partial *η*^2^ = 0.697). No significant differences were observed between adults who stutter and adults who do not stutter, when controlling for CTOPP BW and CTOPP NWR performances, at the 3-letter length (*t*(22) = 1.997, *p* = 0.058, partial *η*^2^ = 0.153). As seen in Fig 4, adults who stutter were significantly less accurate solving word jumble tasks at the 4-letter, 5-letter, and 6-letter lengths compared to adults who do not stutter.

### Speech reaction time

**Fig 5:**
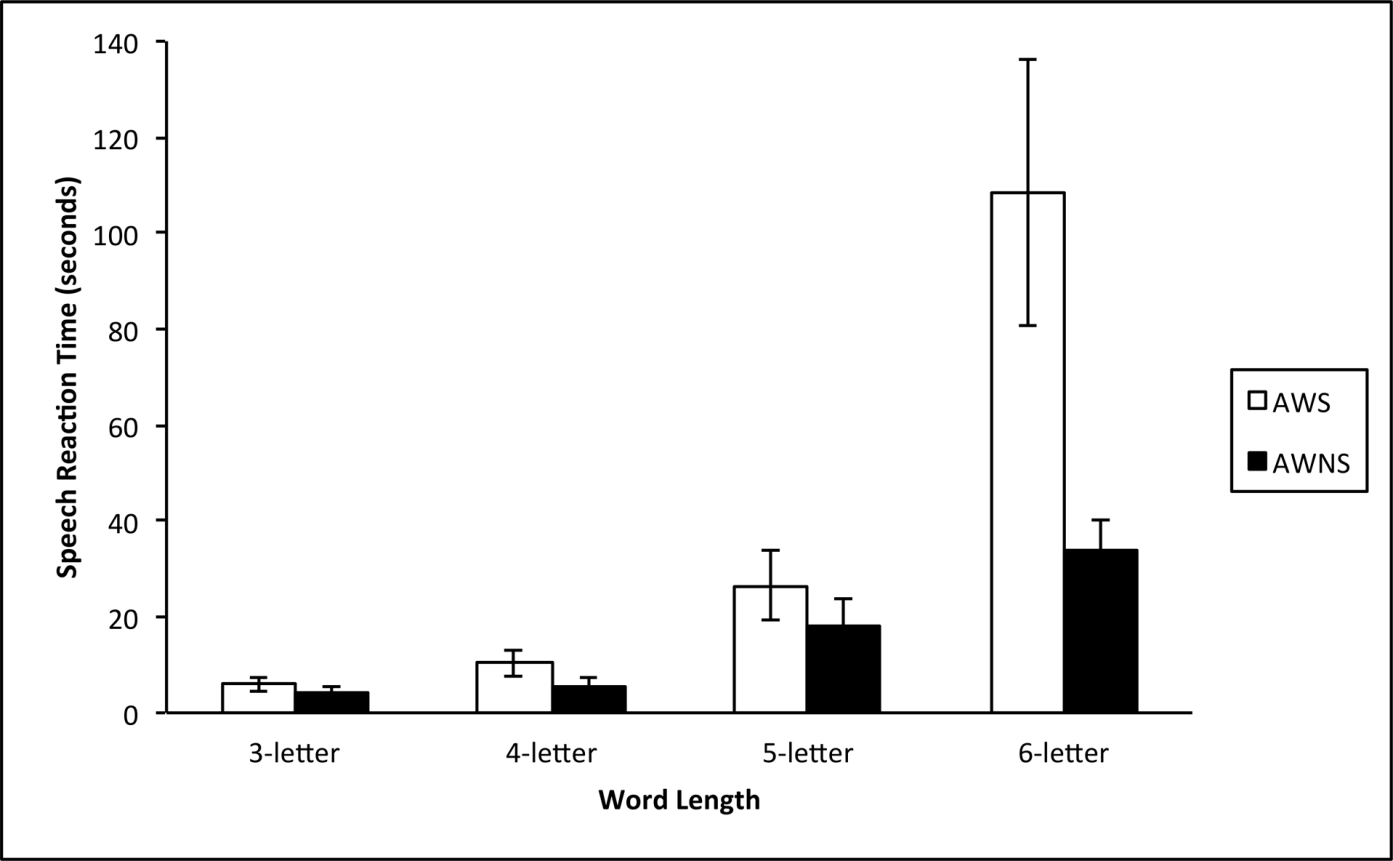
The average speech reaction time in seconds (+/- two standard errors) for each word jumble task across word-length classes for adults who stutter (AWS) and adults who do not stutter (AWNS).

A mixed-model repeated measures ANCOVA was conducted with the between-subjects factor of Talker Group (adults who stutter, adults who do not stutter), a within-subjects factor of word length (3-,4-, 5-,6-letter length), and CTOPP NWR and CTOPP BW performances included as covariates. The dependent variable was the participant’s SRT (in seconds) from the presentation of the scrambled stimuli to the production of an accurate or inaccurate response. SRT for no-response trials was not included in this analysis. Results revealed a significant between-subjects effect for Talker Group *F*(1,22) = 15.984, *p* = 0.001, partial *η*^2^ = 0.421 and a significant interaction between Talker Group and Word Length *F*(3,22) = 16.410, *p* ≤ 0.0001, partial *η*^2^ = 0.427. There was not a significant main effect for Word Length *F*(3,22) = 1.237, *p* = .303, partial *η*^2^ = 0.053. Decomposition of the interaction between Talker Group and Word Length, controlling for CTOPP BW and CTOPP NWR, revealed a significant difference between adults who stutter and adults who do not stutter at the 6-letter word length (*t*(22) = 4.248, *p* ≤ 0.0001, partial *η*^2^ = 0.451). As shown in Fig 5, SRT was significantly slower for adults who stutter than for adults who do not stutter at the longest word length (6-letter).

### Correlation between accuracy and SRT

**Fig 6:**
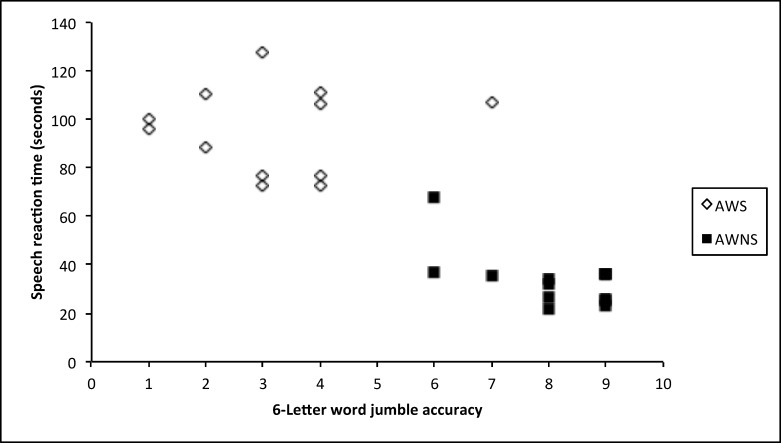
The correlation between accuracy and speech reaction time (in seconds) at the 6-letter word length for adults who stutter (AWS) and adults who do not stutter (AWNS).

An evaluation was made of the linear relationship between SRT and accuracy for each talker group using Pearson’s correlation, controlling for CTOPP BW and CTOPP NWR scores. As depicted in Fig 6, for adults who stutter, at the 6-letter length, the two variables, accuracy and SRT, were not correlated, *r*(10) = 0.06. However, for adults who do not stutter, at the 6-letter word length, accuracy and SRT demonstrated a strong negative relationship *r*(11) = -0.75. In other words, for adults who do not stutter, as accuracy increased, SRT decreased, but for adults who stutter, no relationship between accuracy and SRT was observed. No other meaningful correlations were noted for 3-letter, 4-letter, or 5-letter word lengths for either talker group.

## Discussion

The present study extended investigations of phonological working memory and stuttered speech by using orthographic rather than auditory stimuli. A word jumble task was chosen because the metalinguistic operations underlying successful performance requires an ability to translate sequences of graphemes into their phonemic equivalents, in reiterative fashion, in search of, and eventual access to, a lexical word. Results revealed that adults who stutter were significantly, and progressively, less accurate than adults who do not stutter at solving word jumbles at the 4-letter, 5-letter, and 6-letter lengths, and moreover, demonstrated significantly longer SRT at the longest word length. Thus, rather than seeing a uniform deficiency in phonological processing, processing deficits emerged when the level of cognitive load was increased. This section will focus on potential areas of processing deficiencies in phonological working memory for adults who stutter during their performance on this demanding word jumble task.

### Grapheme to phoneme conversions and phonological encoding: Effect of cognitive load on working memory

Previous research has speculated that phonological encoding differences in persons who stutter may contribute to their difficulty establishing and maintaining fluent speech production, particularly when cognitive load is taxed (e.g., [37], [13]). An example of a cognitive load effect can be seen in a rhyming study conducted by Weber-Fox and colleagues [38]. They compared rhyming performance between adults who do and do not stutter when only visual orthographic input was provided. Participants manually selected a “yes” or “no” button to indicate whether or not the two visually presented words rhymed. Out of the four conditions, the condition considered to be the most phonologically challenging was the only one to reveal talker group differences. In this condition, participants were presented with two words that were orthographically similar but did not rhyme (e.g., “cost” and “most”). Adults who stutter were significantly slower to respond than their fluent peers in this condition. Weber-Fox et al. [38] contend that their findings do not demonstrate that adults who stutter have fundamental phonological processing deficits but that their ability to phonologically encode may be atypically vulnerable when the cognitive demand increases. In the word jumble task, performance differences between adults who do and do not stutter were noted at the longest word lengths (i.e., 5- letter and 6- letter words). The series of phonologically-dependent operations involved in solving a complex word jumble may recruit a high cognitive load and, thus, may have negatively impacted both the accuracy and SRT of adults who stutter in the current study.

### Sub-vocal stimuli manipulations

Nonword repetition and phoneme elision paradigms, which require sub-vocal rehearsal, have demonstrated significant performance differences in adults who stutter relative to adults who do not stutter (e.g., [8], [12], [13]). Such tasks heavily depend on sub-vocal rehearsal (i.e., keeping the stimuli in short term memory storage In the present task, sub-vocal rehearsal is not required as defined by Baddeley [24] because the participant has continual visual access to the word as he/she is attempting to unscramble it. However, the participant is required to nonvocally manipulate the stimulus in a trial and error fashion to arrive at a lexically appropriate solution. The numerous trial and error rearrangements, including assessing if the reordered letter strings match a lexical word, provides a robust processing load on phonological encoding and rehearsal networks. A similar cognitive load effect was seen in Byrd et al. [13]. Adults who stutter, relative to fluent controls, demonstrated significant difficulty manipulating a novel phonological string, even when vocal output was not required. Unlike the visual stimuli used in the present study, Byrd and colleagues [13] provided auditory input only. Thus, deficits in covert manipulation of phonological strings, irrespective of modality (i.e. verbal versus visual), appear to be relevant in explain and understanding the performance of adults who stutter on such demanding tasks.

### Lexical access

The nature of the word jumble task requires participants to hold the changing phonological strings in the output buffer while simultaneously accessing the lexicon to determine if their solution to the paradigm is congruent with a real English word. Adults who stutter exhibited both increased SRT and decreased accuracy at the longest (6-letter) word length, whereas, adults who do not stutter demonstrated increased SRTs for 6-letter strings, but also increased accuracy. In other words, adults who do not stutter appeared to present with a speed-accuracy trade-off, such that increased SRT was required in order to produce the accurate solution to the word jumble task at the 6-letter length. Conversely, adults who stutter did not appear to benefit from increased SRT. That is, the slowness of their responses did not correspond with the accuracy of their responses. The absence of the speed-accuracy tradeoff for adults who stutter suggests that lexical access and/or the organization of the mental lexicon may be compromised in adults who stutter. Nevertheless, as has been previously argued by Hakim and Ratner [9] “it is difficult to know whether weaknesses in responding to the tasks reflect difficulty in encoding the input, storing it in memory, or accessing it efficiently (p.194).” More specific to the present study, it is challenging to parse out these individual processes (i.e. grapheme to phoneme conversion, phonological encoding, sub-vocal manipulation of serial ordering of segmental elements, lexical access) that take place nearly simultaneously as part of completing the word jumble paradigm.

### Speech Motor and Additional Considerations

It is also possible that adults who stutter demonstrated a temporal instability in motor programming that resulted in slower and less accurate word jumble responses. Although stuttered responses were excluded from analysis, it is possible that adults who stutter demonstrated increased response times due to avoidance of anticipated stuttering. Previous research using nonword repetition and phoneme elision tasks with adults who stutter lends support to the assumption that there is an interplay between phonological encoding and motor programming (e.g., [13], [39], [40]). Future research could include follow-up questions to determine if participants detected difficulty responding due to anticipated disfluencies and/or if they used any fluency-enhancing strategies that may have impacted the onset of their responses.

Additional research is warranted to better understand the various subsystems involved in motor speech, lexical access, and phonological processing for adults who do and do not stutter. Perhaps, neuroimaging data can be used to further explain the role of phonological processing in stuttered speech.

### Word Jumble Processing in Adults who Stutter: Possible Neural Correlates

In the past decade there has been an upsurge in brain-based studies reporting *structural* (white matter integrity, gray matter volumes), *functional* (overactive and underactive brain regions), and *neuro-pharmacological* (imbalances of D1 and D2 dopamine receptors in the basal ganglia) anomalies in persons who stutter (see Craig-McQuaide et al. [41] for meta-analyses). Collectively, the compromised neural areas, networks, and chemical regulators implicated in stuttering involve cortical/ subcortical areas performing speech motor planning and execution, and their subsequent integration with a phonological interface—e.g., the ‘speech sound map.’ Neurons in the speech sound map area are hypothesized to encode sound, syllable, and word sequences to be articulated [42]. This section of the discussion will provide an overview of diffusion tractography studies in persons who stutter, focusing on region-to-region white matter connectivity within the dorsal stream [43]. The dorsal stream contains the superior longitudinal fasciculus and the arcuate fasciculus. Of particular importance to the present study, these pathways are thought to sustain the “dorsal phonological route underlying grapheme-phoneme decoding” ([44], p. 935).

To provide a framework for interpreting neuroimaging studies completed with persons who stutter, it is critical to first understand the general assumptions underlying the relationship between the dorsal stream and phonological processing. Saygin et al. [23] reported composite scores on measures of phonological awareness (elision, blending, nonword repetition, letter knowledge) positively correlated with white matter volume of the left arcuate fasciculus in typically developing children. With that in mind, it is interesting to note that Chang et al. [18] reported lower tract densities in adults who stutter in connections between Broca’s area and both left primary motor cortex and premotor cortex. Chang and Zhu [19] also reported lower fractional anisotrophy (FA) values in children who stutter in basal ganglia-to-thalamus-to-cortex loops as well as connections between posterior superior temporal regions and the left inferior frontal gyrus. While FA is a fairly non-specific biomarker of fiber neuropathology, lower FA values are associated with slower speed and reduced quality of signal transmissions, or less-than-ideal synchronization of signals between relevant brain regions [21].

A more recent investigation by Connally et al. [21] reported lower FA values in the arcuate fasciculus bilaterally, as well as an inverse correlation between FA value and stuttering severity in the left angular gyrus. Cai et al. [17] established a region-to-region connectivity matrix in adults who stutter showing a wide array of inferior connections in several regions of left peri-Rolandic sensorimotor cortex and left premotor areas, most notably between left ventral premotor cortex and mid-regions of primary motor cortex. Correlational analyses between severity of stuttering and white matter connectivity in the speech network revealed that all significant correlations were negative—low FA values correlated with high stuttering severity.

Additionally, Cieslak et al. [20] reported three white matter tracts that significantly differentiated adults who stutter from fluent controls: (1) the left arcuate fasciculus connecting the left inferior temporal gyrus to the left insular cortex was missing 39% of the ‘tract’ volume, (2) the right arcuate fasciculus was also missing streamlines (38% of the ‘tract’) connecting inferior temporal gyrus to the supramarginal gyrus and surprisingly, (3) a ‘novel’ left temporo-striatal tract connecting left inferior temporal gyrus to the left accumbens area of the basal ganglia was found only in adults who stutter (7 of the 8 participants who stutter). This white matter pathway overlaps with the uncinate fasciculus, which has been functionally linked to “auditory working memory/sound recognition” ([45], p. 3538). Thus, findings from diffuse tensor tractography studies provide a neural basis for behavioral differences in phonological working memory for persons who stutter relative to fluent controls.

Additional supportive evidence of network dysfunction in frontal speech motor areas in adults who stutter has been reported in a whole-head magnetoencephalography (MEG) mapping study [22] using a delayed reading (isolated nouns) paradigm. The temporal patterning of cortical activations during single word reading in adults who stutter were found to be reversed relative to fluent controls. Within the initial 400 milliseconds after seeing a word, timing order of cortical activation in fluent controls proceeded from left inferior frontal cortex (articulatory programming) to left lateral central sulcus and dorsal premotor cortex (motor preparation); in contrast, adults who stutter showed an early left motor cortex activation followed by a long delayed left inferior frontal signal. This implies that the functional synchronization between preparation and execution of an articulatory motor sequence in persons who stutter is out of phase. High temporal resolution methodologies, such as MEG recordings, are essential to further identify subtle serial ordering anomalies in activating the speech production system. Taken together, behavioral data from the current study and previously reported neuroimaging results support the idea that differences in phonological processing and/or lexical access may contribute to the persistence and maintenance of stuttered speech production.

## Conclusion

Through the use of behavioral tasks such as nonword repetition and phoneme elision, researchers have demonstrated that phonological working memory may contribute to stuttered speech production (e.g., [5]; cf., [7]). The current investigation extended past research by taxing phonological working memory using phonologically scrambled visual stimuli (i.e. word jumble task). As the cognitive load increased, adults who stutter attempted to solve significantly fewer word jumble tasks compared to fluent controls. Of the trials attempted, adults who stutter were significantly less accurate in their completion of word jumble tasks at the 4-letter, 5-letter, and 6-letter lengths compared to adults who do not stutter. Furthermore, adults who stutter demonstrated increased SRT at the 6-letter word length as compared to fluent controls. Additionally, adults who do not stutter demonstrated a significant negative correlation between accuracy and SRT at the longest word length; however, adults who stutter did not appear to present with this speed-accuracy trade-off. These results may be interpreted to further support the notion that differences in various aspects of phonological working memory, including visual-to-sound conversions, lexical access, and sub-vocal manipulations, may uniquely compromise the speech fluency of adults who stutter. Various neural underpinnings for both phonological processing deficits and speech motor control deficits may overlap and be responsible for the “on-off” fluency problems characterizing stuttering.

## Supporting Information

S1 Data Accuracy and speech reaction time data for adults who do and do not stutter. (XLSX)
